# Supplementary material for: A Summer of Cyanobacterial Blooms in Belgian Waterbodies: Microcystin Quantification and Molecular Characterizations
Source: Toxins (Basel). 2022 Jan 16;14(1):61. doi: 10.3390/toxins14010061 (PMC8780180; doi:10.3390/toxins14010061)
Supplement: Supplementary file 1 [file toxins-14-00061-s001.zip › toxins-1494079-supplementary.pdf]

toxins

Supplementary

A Summer of Cyanobacterial Blooms in Belgian Waterbodies: Microcystin Quantification and Molecular Characterizations

Wannes Hugo R. Van Hassel [1,2,*], Mirjana Andjelkovic [3], Benoit Durieu [2], Viviana Almanza Marroquin [4], Julien Masquelier [1], Bart Huybrechts [1,†] and Annick Wilmotte [2]

[1] Toxins unit, Organic Contaminants and Additives, Sciensano, Rue Juliette Wytsmanstraat 14, 1050 Brussels, Belgium; Julien.Masquelier@sciensano.be (J.M); Bart.Huybrechts@sciensano.be (B.H.)
[2] InBios-Centre for Protein Engineering, Department of Life Sciences, University of Liège, Allée du six Août 11, 4000 Liège, Belgium; benoit.durieu@uliege.be (B.D.); awilmotte@uliege.be (A.W.)
[3] Risk and-Health Impact Assessment, Sciensano, Rue Juliette Wytsmanstraat 14, 1050 Brussels, Belgium; Mirjana.Andjelkovic@sciensano.be
[4] Phytoplankton and Phytobenthos laboratory, EULA Center, University of Concepcion, Barrio Universitario box 160, 3349001 Concepcion, Chile; valmanza@udec.cl
* Correspondence: Wannes.Vanhassel@sciensano.be
† The author has passed away.

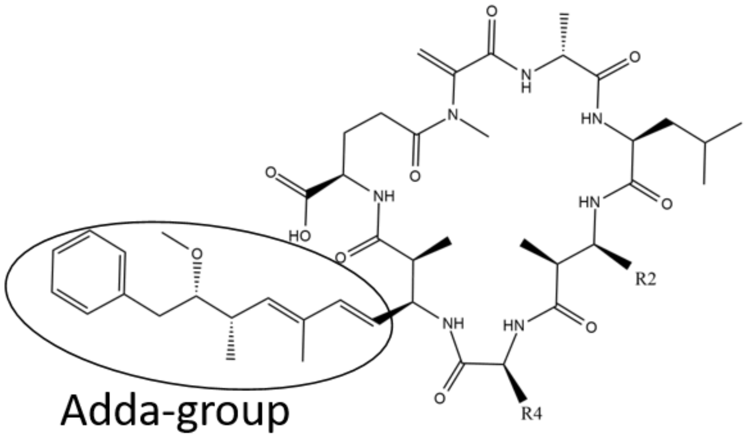

Figure S1. Microcystin core structure with annotated adda-group.

.

Table S1. Overview of experimental data for the water samples, concentrations of MC congeners and total microcystin ($\mu g\ L^{-1}$), presence of genes coding for 16S rRNA and *mcyE*. molecular identification, coverage and identity % of the most similar hit by BLAST and primer used for Sanger sequencing. The first three characters of the samples annotation indicate the sample location, while the last two numbers indicate the week of the year when the sample was collected. Samples are grouped by origin, Flanders. Brussels and Wallonia. N.A. indicates samples for which no PCR was performed. “-“ represent samples were no visible bands were obtained after gel electrophoresis of the PCR products or sequencing results were off too pore a quality to provide a reliable result. “?” represent inconclusive PCR results where the band was visible but was too faint or not at the correct height. The asterisk * indicates that the sequence was obtained on the basis of a PCR reaction produced with the primer pair 359F-781R, whereas the others sequences were obtained with 359F-32S30R. “^{i}” denotes the samples that were analyzed using Illumina technology.

Samples	MC-RR $\mu g\ L^{-1}$	MC-LA $\mu g\ L^{-1}$	MC-LF $\mu g\ L^{-1}$	MC-LR $\mu g\ L^{-1}$	MC-LY $\mu g\ L^{-1}$	MC-LW $\mu g\ L^{-1}$	MC-YR $\mu g\ L^{-1}$	MC-WR $\mu g\ L^{-1}$	Total microcystin $\mu g\ L^{-1}$	16s rRNAs	Fresh biomass (10^{-6} g L^{-1})	*mcyE*	Molecular identification (genus)	coverage (%)	Per. Identity (%)	primer
**AN1.32**	0.44	0.00	0.00	0.23	0.00	0.00	0.00	0.00	0.67	+	5.4	+	-	-	-	
**AN2.32**	0.00	0.00	8.74	121.77	9.75	2.22	0.00	0.00	142.48	+	15.4	+	*Dolichospermum*	100	100	359F
**AN2.37**	12.77	0.00	<LOQ	6.16	<LOQ	0.00	1.41	0.44	20.78	+	5.6	+	*Synechococcus*	100	99.16	359F
**AN3.37**	0.00	0.00	16.64	105.94	12.75	2.35	0.00	0.00	137.68	+	3.7	+	*Romeria*	99.71	98.65	359F
**GH1.30**	0.00	0.00	0.00	0.00	0.00	0.00	0.00	0.00	0.00	-	4.9	-	-	-	-	
**VL1.32**	206.29	3.73	0.00	75.43	0.13	<LOQ	43.79	24.04	353.40	+	37.7	+	*Microcystis*	100	98.98	781R*
**VL1.36**	357.34	30.31	0.90	196.15	2.66	3.44	103.32	52.56	746.69	+	30.4	+	-	-	-	
**VL2.34**	1565.98	4.28	0.52	538.41	1.16	0.83	241.32	68.41	2420.91	+	13.2	+	*Dolichospermum*	100	100	359F
**VL2.36i**	75.24	0.39	<LOQ	30.00	0.11	0.00	18.45	4.74	128.93	+	2.2	+	*Microcystis*	100	97.81	781R*
**VL2.2.36**	1726.08	7.40	3.02	594.51	3.49	1.60	277.00	185.71	2798.81	+	102.8	+	*Microcystis*	100	99.68	359F*
**VL3.34**	0.16	0.24	0.00	<LOQ	0.00	0.00	0.00	0.27	0.67	+	3.6	+	*Dolichospermum*	100	99.70	359F
**BL1.29**	66.89	7.79	<LOQ	75.62	0.00	0.32	97.30	1.25	249.18	+	4.3	+	*Microcystis*	100	100	781R*
													Synechococcus/Merismopedia	100	98.78	359F

**BL1.30**	143.54	13.45	<LOQ	132.23	0.00	0.59	179.54	3.67	473.01	+	2.3	+	*Microcystis*	100	99.13	781R*
**BL1.35**	132.87	14.41	0.19	179.06	<LOQ	0.56	275.91	2.84	605.85	+	2.9	+	-	-	-	
**BL2.29**	0.00	0.00	0.00	0.68	0.00	0.00	0.00	0.00	0.68	+	3.8	-	-	-	-	
**BL3.30**	0.00	0.00	0.00	0.00	0.00	0.00	0.00	0.00	0.00	+	10.22	-	-	-	-	
**BL4.30**	0.00	0.00	0.00	0.00	0.00	0.00	0.00	0.00	0.00	+	3.5	-	-	-	-	
**BL4.35**	0.11	0.00	0.00	0.00	0.00	0.00	0.00	0.00	0.11	+	1.5	-	*Aphanizomenon*	100	100	359F
**BL5.29i**	0.81	0.00	0.00	0.41	0.00	0.00	0.00	0.00	1.22	+	0.6	+	*Dolichospermum*	100	100	359F
**BL5.35**	0.89	0.00	0.00	0.44	0.00	0.00	<LOQ	0.00	1.33	+	12.2	+	*Dolichospermum*	100	100	359F
**BL6.29**	0.00	0.00	0.00	0	0.00	0.00	0.00	0.00	0.00	+	17.8	-	*Dolichospermum*	100	100	359F
**BL6.35**	0.00	0.00	0.00	0.00	0.00	0.00	0.00	0.00	0.00	+	3.7	+	-	-	-	
**BL7.30**	0.00	0.00	0.00	0.00	0.00	0.00	0.00	0.00	0.00	+	5.5	+	*Dolichospermum*	99.82	99.26	359F
**BL7.35**	0.00	0.00	0.00	<LOQ	0.00	0.00	0.00	0.00	0.00	+	2.2	+	-	-	-	
**BL8.35**	0.51	0.00	0.00	0.39	0.00	0.00	0.16	0.00	1.07	+	2.5	+	*Microcystis*	100	99.65	359F*
**BV1.34**	7.68	0.00	0.00	3.57	0.00	0.00	1.40	<LOQ	12.65	+	1.4	+	*Microcystis*	100	97.94	781R*
**BV2.34**	13.65	0.00	0.00	3.92	0.00	0.00	3.04	1.03	21.63	+	2.3	+	*Microcystis*	100	99.82	23S30R
**BV2.35**	4.53	0.00	0.00	1.70	<LOQ	0.00	0.80	0.31	7.34	+	1.9	+	*Synechococcus*	99.81	96.85	359F
**BV3.35**	1213.11	0.71	0.56	364.91	1.42	0.95	188.58	61.09	1831.32	+	2.5	-	*Phormidium*	100	98.02	359F
**B04.27**	0.65	0.00	0.00	0.41	0.00	0.00	0.00	0.00	1.06	+	2.3	+	*Aphanizomenon*	100	97.97	359F

**B04.28**	0.57	0.00	0.00	0.45	0.00	0.00	0.00	0.00	1.02	+	2.7	+	-	-	-	
**B04.29**	0.25	0.00	0.00	<LOQ	0.00	0.00	0.00	0.00	0.25	+	3.0	+	*Aphanizomenom*	100	100	359F
													Planktothrix	100	98.42	781R*
**B04.30**	<LOQ	0.00	0.00	0.00	0.00	0.00	0.00	0.00	0.00	+	0.9	N.A.	-	-	-	
**B04.31**	0.98	0.00	0.00	0.45	0.00	0.00	0.00	0.00	1.43	+	1.2	+	*Dolichospermum*	100	94.52	359F
**B04.32**	0.72	0.00	0.00	0.49	0.00	0.00	0.00	0.00	1.21	+	2.1	+	-	-	-	
**B04.34**	1.03	0.00	0.00	0.83	0.00	0.00	0.00	0.00	1.86	+	1.8	-	*Aphanizomenon*	99.83	98.09	359F
**B04.35**	0.38	0.00	0.00	0.32	0.00	0.00	0.00	0.00	0.70	+	2.2	?	-	-	-	
**B04.36**	0.19	0.00	0.00	0.22	0.00	0.00	0.00	0.00	0.41	+	2.0	+	-	-	-	
**B04.37**	0.27	0.00	0.00	0.29	0.00	0.00	0.00	0.00	0.56	+	2.1	+	-	-	-	
**E04.27**	0.12	0.00	0.00	<LOQ	0.00	0.00	0.00	0.00	0.12	N.A.	6.1	?	N.A.	N.A.	N.A.	
**E04.28**	0.36	<LOQ	0.00	0.33	0.00	0.00	0.18	0.00	0.87	+	1.7	+	-	-	-	
**E04.29**	0.29	0.00	0.00	0.49	0.00	0.00	0.47	0.00	1.26	+	2.3	+	*Aphanizomenon*	99.85	99.85	359F
**E04.30**	1.93	0.80	0.00	6.96	0.00	0.00	9.12	0.15	18.96	+	1.6	N.A.	-	-	-	
**E04.31**	1.57	0.40	0.00	3.25	0.00	0.00	8.85	0.00	14.07	+	1.4	N.A.	-	-	-	
**E04.32**	0.51	<LOQ	0.00	0.94	0.00	0.00	2.60	0.00	4.05	+	2.2	+	*rhodoplast*	98.9	95.48	781R
**E04.34**	1.71	0.24	0.00	2.31	0.00	0.00	5.13	0.00	9.39	+	2.3	+	*Microcystis*	99	97.00	359F*
**E04.35**	3.17	1.50	0.00	10.64	0.00	0.00	28.02	<LOQ	43.34	+	2.1	?	-	-	-	

**E04.36**	1.70	0.46	0.00	3.21	0.00	0.00	8.41	0.00	13.78	+	2.1	+	*Microcystis*	100	94.00	359F
**E04.37**	0.99	0.21	0.00	1.97	0.00	0.00	4.20	0.00	7.38	N.A.	2.4	+	N.A.	N.A.	N.A.	
**H02.27**	<LOQ	0.00	0.00	0.00	0.00	0.00	0.00	0.00	0.00	+	3.1	?	*Synechococcus*	100	99.59	359F
**H02.28**	0.60	0.00	0.00	<LOQ	0.00	0.00	0.00	0.00	0.60	+	2.2	+	-	-	-	
**H02.29**	0.53	0.00	0.00	0.17	0.00	0.00	0.00	0.00	0.69	+	1.1	N.A.	-	-	-	
**H02.30**	2.18	0.00	0.00	0.87	0.00	0.00	<LOQ	0.00	3.05	+	1.0	N.A.	-	-	-	
**H02.31**	3.16	0.00	0.00	0.71	0.00	0.00	0.18	0.00	4.05	+	0.8	+	*Planktothrix*	99.44	94.99	359F
**H02.32**	3.66	0.00	0.00	0.69	0.00	0.00	<LOQ	0.00	4.35	+	2.1	+	*Aphanizomenon*	100	99.16	359F
**H02.34**	3.32	0.00	0.00	0.69	0.00	0.00	0.18	0.00	4.18	+	1.9	+	-	-	-	
**H02.35**	2.37	0.00	0.00	0.43	0.00	0.00	<LOQ	0.00	2.80	+	1.9	?	*Planktothrix*	100	100	359F
**H02.36**	2.33	0.00	0.00	0.36	0.00	0.00	<LOQ	0.00	2.69	+	3.5	?	*Synechococcus*	99.80	99.45	23S30R
**H02.37**	0.64	0.00	0.00	0.12	0.00	0.00	<LOQ	0.00	0.76	N.A.	3.7	-	-	-	-	
**I01.27**	0.00	0.00	0.00	0.00	0.00	0.00	0.00	0.00	0.00	+	4.6	+	-	-	-	
**I01.28**	0.13	0.00	0.00	<LOQ	0.00	0.00	0.00	0.00	0.13	+	2.7	?	*Cyanobium*	100	98.10	781R
**I01.29**	0.18	0.00	0.00	0.00	0.00	0.00	0.00	0.00	0.18	+	1.3	?	-	-	-	
**I01.30**	0.46	0.00	0.00	0.00	0.00	0.00	0.00	0.00	0.46	+	1.9	N.A.	-	-	-	
**I01.31**	1.26	0.00	0.00	0.24	0.00	0.00	0.15	0.00	1.66	+	0.8	N.A.	-	-	-	
**I01.32**	0.19	0.00	0.00	0.00	0.00	0.00	0.00	0.00	0.19	+	2.3	N.A.	-	-	-	
**I01.34**	0.44	0.00	0.00	0.12	0.00	0.00	0.00	0.00	0.56	+	1.7	+	-	-	-	
**I01.35**	0.15	0.00	0.00	<LOQ	0.00	0.00	0.00	0.00	0.15	+	1.8	+	*Synechococcus*	100	99.81	359F
**I01.36**	0.12	0.00	0.00	0.00	0.00	0.00	0.00	0.00	0.12	+	2.1	-	-	-	-	

**I01.37**	0.44	0.00	0.00	<LOQ	0.00	0.00	0.00	0.00	0.44	+	2.7	+	*Cyanobium*	100	97.30	781R*
**I04.27**	<LOQ	0.00	0.00	0.04	0.00	0.00	0.00	0.00	0.04	+	1.9	+	*Aphanizomenon*	100	99.64	359F
**I04.28**	0.91	0.00	0.00	0.48	0.00	0.00	<LOQ	0.00	1.39	+	1.5	+	*Aphanizomenon*	99.84	98.85	359F
**I04.29**	0.83	0.00	0.00	0.33	<LOQ	0.00	<LOQ	0.00	1.16	+	1.1	+	*Dolichospermum*	100	100	359F
**I04.30**	6.11	0.00	0.00	2.90	0.42	0.00	0.36	0.00	9.79	+	3.3	+	*Dolichospermum*	99.83	99.16	359F
**I04.31**	42.34	0.00	0.00	17.62	3.74	0.52	2.38	<LOQ	66.61	+	2.5	+	*Dolichospermum*	100	99.39	359F
**I04.32**	17.70	0.00	<LOQ	6.92	1.34	0.27	1.73	<LOQ	27.96	+	3.3	+	*Microcystis*	100	99.66	359F*
													Dolichospermum	99.75	98.25	359F
**I04.34**	51.87	<LOQ	0.15	13.95	2.05	0.46	3.44	0.27	72.18	+	3.5	+	*Microcystis*	100	98.65	359F
**I04.35**	106.98	0.86	0.45	36.09	7.50	1.16	7.19	0.32	160.54	+	2.2	+	-	-	-	
**I04.36**	167.40	1.15	0.77	56.88	11.22	1.76	10.59	0.58	250.35	+	2.9	+	*Planktothrix*	99.11	95.63	359F
**I04.37**	140.98	0.73	0.58	43.02	7.40	1.12	7.44	0.28	201.54	+	3.9	+	*Cyanodictyon*	100	98.62.	359F

Table S2. Overview of sampling sites in the different regions, water sample annotation, waterbody type and specific monitoring for cyanobacteria.

Place annotation	Name	Waterbody type	Monitored	Place annotation	Name	Waterbody type	Monitored
	**Wallonia**				**Brussels**		
**IO1**	Lac de Falemprise, Cerfontaine	Recreational lake	Yes	BL1	Boudewijn park fase 1. Jette	Shallow pond	No
**EO4**	Grand large, Mons	Recreational lake	Yes	BL2	Boudewijn park fase 2. Jette	Shallow pond	No
**BO4**	Renipont-Plage, Lasne	Recreational lake	Yes	BL3	Big Mellaert pond. Sint-Pieters-Wolluwe	Shallow pond	No
**HO2**	Sport complex Saint-Leger, Saint-Lèger	Recreational lake	Yes	BL4	Tercoigne laan. Watermaal-Bosvoorde	Shallow pond	No
**IO4**	Lac de Bambois, Fosses-La-Ville	Recreational lake	Yes	BL5	Red cloister 3.Oudergem	Shallow pond	No
	**Flanders**			BL6	Leybeekpond. Watermael-Bosvoorde	Shallow pond	No
**GH1**	Sedimentation pond Gasthuisberg, Leuven	Sedimentation pond	No	BL7	Rue de pecheries.Watermaal-Bosvoorde	Shallow pond	No
**AN1**	Resort De kempen, Mol	Recreational shallow pond	Yes	BL8	Little Mellaert pond. Sint-Pieters-Wolluwe	Shallow pond	No
**AN2**	Hof van Eden, Westerloo	Recreational shallow pond	Yes	BV1	Quai des Péniches. Sint-Jan-Molenbeek	Canal	No
**VL1**	Kleine vijver provincial domain Kessel-Lo, Kessel-Lo	Shallow pond	No	BV2	Qaui de Heembeek Ferry stop. Heembeek	Canal	No
**VL2**	Vossem pond Park, Vossem	Shallow pond	No	BV3	Quai des Charbonnages. Sint-Jan-Molenbeek	Canal	No
**VL3**	Gordaal pond, Tervuren	Shallow pond	No				
**AN3**	Zilverstrand, Mol	Recreational Lake	Yes				

Table S3. Detailed information for taxonomic identification based on BLAST analysis. * Samples for which PCR with different primers gave different dominant taxa.

Place	Sample	qseqid	Accession number	Title	% identity	length	mismatch	gaps	e value	bitscore
**Hof van Eden, Westerloo**	AN2.32	GS20-C085-AWil-20-359F	MH669067	*Dolichospermum circinale* NRERC-107	100	577	0	0	0	1066
	AN2.37	GS20-C088-AWil-26-359F	AM709627	*Synechococcus* sp. PCC 7920	99.165	599	5	0	0	1079
**Zilverstrand, Mol**	AN3.37	GS20-C089-AWil-27-359F	JQ819251	*Romeria* sp. KLL-H-201	98.651	667	5	4	0	1179
**Kleine vijver provincial domain Kessel-Lo, Kessel-Lo**	VL1.32	GS20-C114-AWil-19-781R	LC557463	*Microcystis aeruginosa* NIES-90	98.985	197	2	0	1.71E-93	353
**Vossem pond Park, Vossem**	VL2.34	GS20-C086-AWil-21-359F	FN691909	*Dolichospermum flos-aquae* 04-57	100	427	0	0	0	789
	VL2.36	GS20-C116-AWil-24-781R	LC557463	*Microcystis aeruginosa* NIES-90	97.81	274	4	2	6.57E-129	472
	VL2.36bis	GS20-C064-AWil-25-359F	LC557463	*Microcystis aeruginosa* NIES-90	99.681	313	1	0	2.04E-159	573
**Gordaal Vijver, Tervuren**	VL3.34	GS20-C087-AWil-22-359F	FN691909	*Dolichospermum flos-aquae* 04-57	99.703	673	2	0	0	1232
**Boudewijn park fase 1, Jette**	BL1.29*	GS19-G066-AWil-1-781R	LC557461	*Microcystis aeruginosa* NIES-104	100	341	0	0	1.31E-176	630
	BL1.29*	GS20-C072-AWil-1-359F	MT376739	*Synechococcus* sp. SR-R4S2	98.785	494	6	0	0	880

		GS20-C072-AWil-1-359F	EF088332	*Merismopedia sp.* CENA106	98.785	494	6	0	0	880
	BL1.30	GS19-G068-AWil-5-781R	LC557461	*Microcystis aeruginosa* NIES-104	99.13	345	0	3	1.03E-172	617
**Tercoigne laan, Watermaal-Bosvoorde**	BL4.35	GS20-C080-AWil-11-359F	MN104685	*Aphanizomenon flos-aquae* NRERC-020	100	649	0	0	0	1199
**Red cloister 3, Oudergem**	BL5.29	GS20-C073-AWil-3-359F	KC955174	*Dolichospermum flos-aquae* CHAB 1657	100	509	0	0	0	941
	BL5.35	GS20-C081-AWil-12-359F	MT558568	*Dolichospermum planctonicum* ANCD0809	100	429	0	0	0	793
**Leybeekpond, Watermael-Bosvoorde**	BL6.29	GS20-C074-AWil-4-359F	MT558568	*Dolichospermum planctonicum* ANCD0809	100	540	0	0	0	998
**Rue de pecheries, Watermaal-Bosvoorde**	BL7.30	GS20-C077-AWil-7-359F	LC096267	*Dolichospermum flos-aquae* NIES-75	99.259	540	3	1	0	977
**Little Mellaert pond, Sint-Pieters-Wolluwe**	BL8.35	GS19-G073-AWil-15-359F	LC557461	*Microcystis aeruginosa* NIES-104	99.655	290	0	1	4.1E-146	529
**Quai des Péniches, Sint-Jan-Molenbeek**	BV1.34*	GS19-G072-AWil-8-781R	LC557463	*Microcystis aeruginosa* NIES-90	97.938	194	4	0	1.67E-88	337
	BV1.34*	GS20-C131-AWil-8-23S30R	CP039373	*Synechococcus sp.* CB0101	95.161	62	1	1	2.74E-16	97.1
**Qaui de Heembeek Ferry stop, Heembeek**	BV2.34	GS20-C132-AWil-9-23S30R	AP019314	*Microcystis viridis* NIES-102.	99.818	550	1	0	0	1011

	BV2.35	GS20-C083-AWil-16-359F	MT376735	*Synechococcus sp.* SR-C1	96.846	539	16	1	0	900
**Quai des Charbonnages, Sint-Jan Molenbeek**	BV3.35	GS20-C084-AWil-17-359F	EU196622	*Phormidium* cf. tergestinum Drak	98.023	354	7	0	3E-173	619
**Renipont-Plage, Lasne**	B04.27	GS20-C107-AWil-59-359F	MT443970	*Aphanizomenon gracile* FBCC-A274	97.967	541	11	0	0	939
	B04.29*	GS19-G118-AWil-69-781R	LC455659	*Planktothrix agardhii* NIES-905	98.423	317	5	0	5.78E-155	558
	B04.29*	GS20-C111-AWil-69-359F	MT443970	*Aphanizomenon gracile* FBCC-A274	100	442	0	0	0	817
	B04.31	GS20-C092-AWil-31-359F	MT443970	*Aphanizomenon gracile* FBCC-A274	94.521	292	16	0	9.21E-123	451
		GS20-C092-AWil-31-359F	CP051206	*Dolichospermum flos-aquae* CCAP 1403/13F	94.521	292	16	0	9.21E-123	451
	B04.34	GS20-C104-AWil-53-359F	MT443970	*Aphanizomenon gracile* FBCC-A274	98.087	575	10	1	0	1000
**Grand large, Mons**	E04.29	GS20-C112-AWil-71-359F	MN104685	*Aphanizomenon flos-aquae* NRERC-020	99.846	650	0	1	0	1194
	E04.32	GS20-C121-AWil-46-781R	LT622877	Liagora brachyclada chloroplast isolate J.0126	95.48	177	8	0	2E-72	283
	E04.34	GS19-G105-AWil-51-359F	LC557461	*Microcystis aeruginosa* NIES-104	97.00	309	1	0	4E-145	518

	E04.36	GS20-C067-AWil-43-359F	LC557461	*Microcystis aeruginosa* NIES-104	95.082	297	1	9	4E-120	435
**Sport complex Saint-Leger, Saint-Lèger**	H02.27	GS20-C106-AWil-58-359F	MT376735	*Synechococcus sp.* SR-C1	99.591	489	2	0	0	893
	H02.31	GS20-C093-AWil-32-359F	LC455659	*Planktothrix agardhii* NIES-905	94.986	359	12	6	7.15E-155	558
	H02.32	GS20-C102-AWil-49-359F	LC037454	*Aphanizomenon sp.* NIES-3732	99.161	477	2	2	0	857
	H02.35	GS20-C096-AWil-36-359F	LC455659	*Planktothrix agardhii* NIES-905	100	498	0	0	0	920
	H02.36	GS19-G115-AWil-67-359F	MT376735	*Synechococcus sp.* SR-C1	99.66	296	1	0	5.39E-150	542
**Lac de Falemprise, Cerfontaine**	I01.28	GS20-C124-AWil-65-781R	KM218863	*Cyanobium* sp. UMPCCC 1208	98.099	263	5	0	4.88E-125	459
	I01.35	GS20-C097-AWil-37-359F	MT376735	*Synechococcus sp.* SR-C1	99.815	541	1	0	0	994
	I01.37	GS19-G098-AWil-42-781R	AM710381	*Cyanobium sp.* JJR2A5	97.297	296	8	0	2.55E-138	503
**Lac de Bambois, Fosses-La-Ville**	I04.27	GS20-C105-AWil-57-359F	AJ293129	*Aphanizomenon flos-aquae* PMC9706	99.638	552	2	0	0	1009
	I04.28	GS20-C109-AWil-62-359F	EU078530	*Aphanizomenon gracile* LMECYA 9	98.851	609	6	1	0	1085

	I04.29	GS20-C113-AWil-72-359F	AP018316	*Dolichospermum compactum* NIES-806	100	588	0	0	0	1086
	I04.30	GS20-C090-AWil-29-359F	AP018316	*Dolichospermum compactum* NIES-806	99.157	593	4	1	0	1066
	I04.31	GS20-C091-AWil-30-359F	AP018316	*Dolichospermum compactum* NIES-806	99.391	657	4	0	0	1192
	I04.32*	GS19-G101-AWil-47-359F	LC557461	*Microcystis aeruginosa* NIES-104	99.656	291	1	0	3.18E-147	532
	I04.32*	GS20-C101-AWil-47-359F	GU197633	*Dolichospermum circinale* CHAB233	98.25	400	6	1	0	699
	w19.I04.34	GS20-C069-AWil-52-359F	LC557461	*Microcystis aeruginosa* NIES-104	98.653	297	1	3	1.96E-144	523
	I04.36	GS20-C100-AWil-45-359F	LC455659	*Planktothrix agardhii* NIES-905	95.633	229	5	5	3.3E-96	363
	I04.37	GS20-C098-AWil-39-359F	AM710382	*Cyanodictyon* sp. JJCD	98.618	217	3	0	6.72E-103	385

Table S4. Number of reads and BLAST analysis of the representative sequence of the OTUs obtained with the Illumina amplicon sequencing.

#OTU ID	BL5.29. Red Cloister Oudergem	VL1.36. Kessel-Lo Provincial Domain	Accession number of the most similar cultured hit	Name of the most similar cultured hit	% identity	length	mismatch	gaps	e value	bitscore
OTU_72	5027	12	AY701560	*Anabaena ellipsoides* Ana HB	100	362	0	0	0	669
OTU_28	1517	37200	LC557463	*Microcystis aeruginosa* NIES-90	100	362	0	0	0	669
OTU_231	318	230	HE975013	*Aphanizomenon flos-aquae* CCAP 1401/7	100	362	0	0	0	669
OTU_346	185	0	AB610891	*Synechococcus sp*. Suigetsu-CG2	97.79	362	8	0	6E-175	625
OTU_559	127	1	EU078524	*Anabaena spiroides* LMECYA 161	99.448	362	2	0	0	658
OTU_582	76	1	LC319779	*Dolichospermum sp*. NIES-1951	99.171	362	3	0	0	652
OTU_678	61	0	FN691909	*Dolichospermum flos-aquae* 04-57	100	362	0	0	0	669
OTU_537	50	41	CP003495	*Cyanobium gracile* PCC 6307	100	362	0	0	0	669
OTU_649	25	0	AM710380	*Cyanobium sp*. JJM10D5	99.724	362	1	0	0	664
OTU_627	20	0	GU936926	Chlorophyta symbiont of *Lubomirskia* sp. isolate R53	100	362	0	0	0	669
OTU_4	0	7	LT634149	*Timaviella circinata* GR4	99.448	362	2	0	0	658
OTU_5	0	7	MH688842	*Hormoscilla* cf. pringsheimii Us-s-6-2	98.619	362	5	0	6E-180	641
OTU_6	0	7	KM052844	*Leptolyngbya sp*. LCR-CYANT35	99.724	362	1	0	0	664

Table S5. Validation results for UHPLC-MS/MS method quantification method of 8 MCs and Nodularin in filtered cyanobacterial biomass. The different validation concentration levels Limit of detection (LOD) was set at lowest tested concentration where signal to noise ratio was higher than 3. Limit of Quantification (LOQ) was selected as lowest concentration for which the method was validated. Signal to noise ratio for LOQ should be above 20. Additionally, values for recovery, repeatability, reproducibility, measurement uncertainty (MU) and R^2 of the linear curve are presented.

Toxins	Spiked Concentration	Recovery (%)	Repeatability (%)	Reproducibility (%)	MU (%)	Average S/N LOD (5.63 µg kg^{-1})	Average S/N LOQ (12.5 µg kg^{-1})	R^2
**MC-RR**	12.5 µg kg-1	88.00%	4.18%	21.94%	43.88%	531.24	765.34	0.9994
	50 µg kg-1	89.00%	10.54%	10.54%	21.07%			
	125 µg kg-1	92.00%	5.82%	15.65%	31.29%			
	Average	89.67%	6.85%	16.04%	32.08%			
**MC-LA**	12.5 µg kg-1	100.00%	5.67%	14.40%	28.80%	69.07	134.83	0.9992
	50 µg kg-1	89.00%	5.46%	7.71%	15.41%			
	125 µg kg-1	101.00%	2.45%	6.81%	13.62%			
	Average	96.67%	4.53%	9.64%	19.28%			
**MC-LF**	12.5 µg kg-1	95.00%	3.12%	18.51%	37.02%	64.03	139.69	0.9992
	50 µg kg-1	88.00%	5.85%	9.88%	19.76%			
	125 µg kg-1	101.00%	1.65%	3.67%	7.33%			
	Average	94.67%	3.54%	10.68%	21.37%			
**MC-LR**	12.5 µg kg-1	99.00%	4.71%	8.81%	17.62%	116.82	265.64	0.9988
	50 µg kg-1	92.00%	6.53%	7.04%	14.09%			
	125 µg kg-1	101.00%	1.45%	3.21%	6.43%			
	Average	97.33%	4.23%	6.36%	12.71%			
**MC-LY**	12.5 µg kg-1	96.00%	4.45%	13.11%	26.21%	51.57	78.84	0.9992
	50 µg kg-1	88.00%	3.12%	8.29%	16.57%			
	125 µg kg-1	100.00%	1.92%	4.76%	9.53%			
	Average	94.67%	3.16%	8.72%	17.44%			
**MC-LW**	12.5 µg kg-1	100.00%	6.63%	11.12%	22.23%	42.89	92.86	0.9989
	50 µg kg-1	90.00%	3.39%	11.93%	23.87%			
	125 µg kg-1	102.00%	2.21%	6.26%	12.51%			
	Average	97.33%	4.08%	9.77%	19.54%			
**MC-YR**	12.5 µg kg-1	100.00%	6.38%	9.52%	19.05%	75.26	139.15	0.9987
	50 µg kg-1	94.00%	4.55%	4.55%	9.10%			

	125 µg kg-1	98.00%	3.09%	3.58%	7.15%			
	Average	97.33%	4.67%	5.88%	11.77%			
**MC-WR**	12.5 µg kg-1	96.00%	9.69%	9.69%	19.38%	83.91	148.68	0.999
	50 µg kg-1	91.00%	9.57%	12.86%	25.73%			
	125 µg kg-1	100.00%	3.73%	4.90%	9.81%			
	Average	95.67%	7.67%	9.15%	18.31%			
**SUM**	112.5 µg kg-1	96.00%	3.24%	11.27%	22.55%	N.A.	N.A.	N.A.
	450 µg kg-1	91.00%	2.84%	5.46%	10.91%			
	1125 µg kg-1	99.00%	2.09%	3.76%	7.52%			
	Average	95.33%	2.72%	6.83%	13.66%			

Table S6. Results of the ion ratio for the validation of the UHPLC-MS/MS method quantification method of 8 MCs and Nodularin in filtered cyanobacterial biomass. Acceptation criteria are based on European Decision 2002/EC/657.

**Toxin**	**MC-RR**	**NOD**	**MC-LA**	**MC-LF**	**MC-LR**	**MC-LY**	**MC-LW**	**MC-YR**	**MC-WR**
Average ion ratio	13.37%	46.65%	51.91%	41.51%	31.66%	45.82%	49.20%	33.21%	40.21%
Standard Deviation ion ratio	0.39%	2.08%	7.59%	2.43%	1.26%	3.68%	6.15%	3.19%	8.11%

Table S7. Detection results for 6 additional microcystin congeners with a limit of detection (LOD) at 0.1 μg L^{-1}. Not detected is abbreviated by "n.d.". Detected MCs are annotated as > LOD.

Samples	MC-HtyR	dm MC-LR/D-asp MC-LR	D-asp-Dhb MC-RR/dm MC-RR	MC-HilR
AN1.32	n.d.	n.d.	> LOD	n.d.
AN2.32	n.d.	> LOD	> LOD	> LOD
AN2.37	> LOD	> LOD	> LOD	> LOD
AN3.37	n.d.	> LOD	n.d.	> LOD
GH1.30	n.d.	n.d.	n.d.	n.d.
VL1.32	n.d.	> LOD	> LOD	> LOD
VL1.36	n.d.	> LOD	> LOD	> LOD
VL2.34	n.d.	> LOD	> LOD	> LOD
VL2.36i	n.d.	> LOD	> LOD	> LOD
VL2.2.36	n.d.	> LOD	> LOD	> LOD
VL3.34	n.d.	n.d.	n.d.	n.d.
BL1.29	> LOD	> LOD	> LOD	> LOD
BL1.30	> LOD	> LOD	> LOD	> LOD
BL1.35	> LOD	> LOD	> LOD	> LOD
BL2.29	n.d.	> LOD	n.d.	n.d.
BL3.30	n.d.	n.d.	n.d.	n.d.
BL4.30	n.d.	n.d.	n.d.	n.d.
BL4.35	n.d.	n.d.	n.d.	n.d.
BL5.29i	n.d.	n.d.	n.d.	n.d.
BL5.35	n.d.	n.d.	> LOD	n.d.
BL6.29	n.d.	n.d.	n.d.	n.d.
BL6.35	n.d.	n.d.	n.d.	n.d.
BL7.30	n.d.	n.d.	n.d.	n.d.
BL7.35	n.d.	n.d.	n.d.	n.d.
BL8.35	n.d.	n.d.	> LOD	n.d.
BV1.34	n.d.	> LOD	> LOD	> LOD
BV2.34	> LOD	> LOD	> LOD	> LOD
BV2.35	> LOD	> LOD	> LOD	> LOD
BV3.35	n.d.	> LOD	> LOD	> LOD

B04.27	n.d.	n.d.	> LOD	n.d.
B04.28	n.d.	n.d.	> LOD	n.d.
B04.29	n.d.	n.d.	> LOD	n.d.
B04.30	n.d.	n.d.	> LOD	n.d.
B04.31	n.d.	n.d.	> LOD	n.d.
B04.32	n.d.	> LOD	> LOD	n.d.
B04.34	n.d.	n.d.	> LOD	n.d.
B04.35	n.d.	n.d.	> LOD	n.d.
B04.36	n.d.	n.d.	> LOD	n.d.
B04.37	n.d.	n.d.	> LOD	n.d.
E04.27	n.d.	n.d.	> LOD	n.d.
E04.28	n.d.	> LOD	> LOD	n.d.
E04.29	n.d.	> LOD	> LOD	n.d.
E04.30	> LOD	> LOD	> LOD	> LOD
E04.31	n.d.	> LOD	> LOD	> LOD
E04.32	n.d.	> LOD	> LOD	n.d.
E04.34	n.d.	> LOD	> LOD	> LOD
E04.35	> LOD	> LOD	> LOD	> LOD
E04.36	> LOD	> LOD	> LOD	> LOD
E04.37	n.d.	> LOD	> LOD	> LOD
H02.27	n.d.	n.d.	> LOD	n.d.
H02.28	n.d.	n.d.	> LOD	n.d.
H02.29	n.d.	n.d.	> LOD	n.d.
H02.30	n.d.	> LOD	> LOD	n.d.
H02.31	> LOD	> LOD	> LOD	n.d.
H02.32	n.d.	> LOD	> LOD	n.d.
H02.34	n.d.	> LOD	> LOD	n.d.
H02.35	n.d.	> LOD	> LOD	n.d.
H02.36	n.d.	> LOD	> LOD	n.d.
H02.37	n.d.	> LOD	> LOD	n.d.
I01.27	n.d.	n.d.	n.d.	n.d.
I01.28	n.d.	n.d.	n.d.	n.d.

I01.29	n.d.	n.d.	n.d.	n.d.
I01.30	n.d.	n.d.	n.d.	n.d.
I01.31	n.d.	n.d.	> LOD	n.d.
I01.32	n.d.	n.d.	n.d.	n.d.
I01.34	n.d.	n.d.	> LOD	n.d.
I01.35	n.d.	n.d.	> LOD	n.d.
I01.36	n.d.	n.d.	> LOD	n.d.
I01.37	n.d.	n.d.	> LOD	n.d.
I04.27	n.d.	n.d.	n.d.	n.d.
I04.28	> LOD	> LOD	> LOD	n.d.
I04.29	n.d.	> LOD	> LOD	n.d.
I04.30	n.d.	> LOD	> LOD	n.d.
I04.31	n.d.	> LOD	> LOD	> LOD
I04.32	n.d.	> LOD	> LOD	> LOD
I04.34	n.d.	> LOD	> LOD	> LOD
I04.35	n.d.	> LOD	> LOD	> LOD
I04.36	n.d.	> LOD	> LOD	> LOD
I04.37	n.d.	> LOD	> LOD	> LOD

Table S8. Overview of the single sequences amplified by the Sanger method used for taxonomic identification based on BLAST analysis.

Sample number and primer number	Genus name	Sanger sequence
AN2.32_359F	*Dolichospermum*	AGGCTCTTGGGTTGTAAACCTCTTTTCTCAGGGAAGAAAAAAATGACGGTACCTGAGGAATAAGCATCGG<br>CTAACTCCGTGCCAGCAGCCGCGGTAATACGGAGGATGCAAGCGTTATCCGGAATGATTGGGCGTAAAG<br>GGTCCGCAGGTGGCATTGTAAGTCTGCTGTTAAAGAGTCTAGCTCAACTAGATAAAAGCAGTGGAAACTA<br>CAAAGCTAGAGTTTGGTCGGGGCAGAAGGAATTCCTGGTGTAGCGGTGAAATGCGTAGATATCAGGAAG<br>AACACCAGTGGCGAAGGCGTTCTGCTAGGCCGAGACTGACACTGAGGGACGAAAGCTAGGGGAGCGAAT<br>GGGATTAGATACCCCAGTAGTCCTAGCCGTAAACGATGGATACTAGGCGTAGCTCGTATCGACCCGAGCT<br>GTGCCGGAGCTAACGCGTTAAGTATCCCGCCTGGGGAGTACGCAGGCAACTGTGAAACTCAAAGGAATT<br>GACGGGGGCCCGCACAAGCGGTGGAGTATGTGGTTTAATTCGATGCAACGCGAAGAACCTTACCAAGGC<br>TTGACATGTCACGAATCCTGTG
AN2.37_359F	*Synechococcus*	TTCCGTGCCAGCAGCCGCGGTAATACGGAGTGGCAAGCGTTATCCGGAATTATTGGGCGTAAAGCGTCC<br>GCAGGCGGCCTTGTAAGTCTGTCGTTAAAGCGTGGAGCTCAACTCCATTTAAGCGATGGAAACTACAAGG<br>CTGGAGTGTGGTAGGGGCAGAGGGAATTCCCGGTGTAGCGGTGAAATGCGTAGATATCGGGAAGAACAC<br>CAGTGGCGAAGGCGCTCTGCTGGGCCATAACTGACGCTCATGGACGAAAGCCAGGGGAGCGAAAGGGAT<br>TAGATACCCCTGTAGTCCTGGCCGTAAACGATGAACACTAGGTGTCGGGGGAATCGACCCCCTCGGTGTC<br>GTAGCCAACGCGTTAAGTGTTCCGCCTGGGGAGTACGCACGCAAGTGTGAAACTCAAAGGAATTGACGG<br>GGGCCCGCACAAGCGGTGGAGTATGTGGTTTAATTCGATGCAACGCGAAGAACCTTACCAGGGCTTGACA<br>TGCTGCGAATCCCCTGGAAACGAGGGAGTGCCTTCGGGAGCGCAGAGACAGGTGGTGCATGGCTGTCGTC<br>AGCTCGTGTCGTGAGATGTTGGGTTAAGTCCCGCAACGAGCG
AN3.37_359F	*Romeria*	AGGAGGCCTTAGGGTTGTAAACCTCTTTTCTCTGGGAAGAAGAACTGACGGTACCAGAGGAATAAGCCTC<br>GGCTAACTCCGTGCCAGCAGCCGCGGTAAGACGGAGGAGGCGAGCGTTATCCGGAATTATTGGGCGTAA<br>AGCGTCCGCAGGCGGTTTATCAAGTCTGCTGTCAAAGACTACAGCTTAACTGTGGGCAGGCAGTGGAAAC<br>TGATGAACTAGAGAGCGGTAGGGGTAGAGGGAATTCCCGGTGTAGCGGTGAAATGCGTAGATATCGGGA<br>AGAACACCAGTGGCGAAGGCGCTCTACTGGGCCGTTACTGACGCTGAGGGACGAAAGCTAGGGGAGCGA<br>AAGGGATTAGATACCCCTGTAGTCCTAGCTGTAAACGATGGATACTAGGTGTTGGGCGTATCGACCCGTC<br>CAGTACCGTAGCTAACGCGTTAAGTATCCCGCCTGGGGAGTACGCACGCAAGTGTGAAACTCAAAGGAA<br>TTGACGGGGGCCCGCACAAGCGGTGGAGGATGTGGTTTAATTCGATGCAACGCGAAGAACCTTACCAAG<br>GCTTGACATCCTGCGAATCCTTCAGAGATGAGGGAGTGCCTTCGGGAGCGCAGAGACAGGTGGTGCATGG<br>CTGTCGTCAGCTCGTGTCGTGAGATGTTGGGTTAAGTC
VL1.32_781R	*Microcystis*	GCCACCGATGTTCTTCCCAATCTCTACGCATTTCACCGCTACACTGGGAATTCCTGCTACCCCTACTGCTCT<br>CTAGTCTGCCAGTTTCCACCGCCTTTATGTCGTTAAGCAACCTGATTTGACAGCAGACTTGGCTGACCACC<br>TGCGGACGCTTTACGCCCAATAATTCCGGATAACGCTTGCCTCCCCCGTATTACCGCGGCTGCTGGCACGG<br>AGTTAGCCGATGCTGATTCCTCAAGTACCGTCA

VL2.34_359F	*Dolichospermum*	TCTGAAGGAAGCATGACGAGGAAATCACTACGCTCTGGGGTTGTAGCCTCTTTTCTAATGGAAGAAGTGC<br>TGTTGGTACCTGGGTGTTAATCATCGTCTAACTCCGGGGAAGCTGCCGCGGTCTTACGGAGGATGCAAGC<br>GTTATCCGGAATGATTGGGCGTAAAGGGTCCGCAGGTGGCATTGAAAGTCTGCTGTTCAGAGGTTGGGTC<br>AACCAAATAAGAGCAGTGGAAACTACAAAGCTAGAGTTTGGTCGGGGCAGAGGGAATTCCTGGTGTAGC<br>GGTGAAATGCGTAGATATCAGGAAGAACACCAGTGGCGAAGGCGCTCTGCTAGGCCGAGACTGACACTG<br>AGGGACGAAAGCTAGGGGAGCGAATGGGATTAGATACCCCAGTAGTCCTAGCCGTAAACGATGGATACT<br>AGGCGTAGCTCGTATCGACCCGAGCTGTGCCGGAGCTAACGCGTTAAGTATCCCGCCTGGGGAGTACGCA<br>GGCAACTGTGAAACTCAAAGGAATTGACGGGGGCCCGCACAAGCGGTGGAGTATGTGGTTTAATTCGAT<br>GCAACGCGAAGAACCTTACCAAGGCTTGACATGTCACGAATCCTGTAGAAATATAGGAGTGCCTTCGGGA<br>GCGTGAACACAGGTGGTGCATGGCTGTCGTCAGCTCGTGTCGTGAGATGTTGGGTTAAGTC
VL2.36.bis_359F	*Microcystis*	TCTGACGGTACTTGAGGAATCAGCCTCGGCTAACTCCGTGCCAGCAGCCGCGGTAATACGGGGGAGGCA<br>AGCGTTATCCGGAATTATTGGGCGTAAAGCGTCCGCAGGTGGTCAGCCAAGTCTGCTGTCAAATCAGGTT<br>GCTTAACGACCTAAAGGCGGTGGAAACTGGCAGACTAGAGAGCAGTAGGGGTAGCAGGAATTCCCAGTG<br>TAGCGGTGAAATGCGTAGAGATTGGGAAGAACATCGGTGGCGAAAGCGTGCTACTGGGCTGTATCTGAC<br>ACTCATGGACGAAAGCTAGGGGAGCGAAAGGGATTAA
VL2.36_781R	*Microcystis*	TCTCTACGCATTTCACCGCTACACTGGGAATTTCCTGCTGCCCCTACTGCTCTCTAGTCTGCCAGTTTCCAC<br>CGCCTTTAGGTCGTTAAGCAACCTGATTTGACAGCAGACTTGGCTGACCACCTGCGGACGCTTTACGCCCC<br>AATAATTCCGGATAACGCTTGCCTCCCCCGTATTACCGCGGCTGCTGGCACGGAGTTAGCCGATGCTGATT<br>CCTCAAGTACCGTC
VL3.34_359F	*Dolichospermum*	TCTTGGGTTGTAAACCTCTTTTCTCAGGGAAGAACAGAATGACGGTACCTGAGGAATAAGCATCGGCTAA<br>CTCCGTGCCAGCAGCCGCGGTAATACGGAGGATGCAAGCGTTATCCGGAATGATTGGGCGTAAAGGGTCC<br>GCAGGTGGCATTGAAAGTCTGCTGTTAAAGAGTTTGGCTCAACCAAATAAGAGCAGTGGAAACTACAAA<br>GCTAGAGTTTGGTCGGGGCAGAGGGAATTCCTGGTGTAGCGGTGAAATGCGTAGATATCAGGAAGAACA<br>CCAGTGGCGAAGGCGCTCTGCTAGGCCGAGACTGACACTGAGGGACGAAAGCTAGGGGAGCGAATGGG<br>ATTAGATACCCCAGTAGTCCTAGCCGTAAACGATGGATACTAGGCGTAGCTCGTATCGACCCGAGCTGTG<br>CCGGAGCTAACGCGTTAAGTATCCCGCCTGGGGAGTACGCAGGCAACTGTGAAACTCAAAGGAATTGAC<br>GGGGGCCCGCACAAGCGGTGGAGTATGTGGTTTAATTCGATGCAACGCGAAGAACCTTACCAAGGCTTGA<br>CATGTCACGAATCCTGTAGAAATATAGGAGTGCCTTCGGGAGCGTGAACACAGGTGGTGCATGGCTGTCG<br>TCAGCTCGTGTCGTGAGATGTTGGGTTAAGTCCCGCAACGAGCGCAAC
BL1.29_359F	*Synechococcus/ Merismopedia*	AGCCGCGGTAATACGGGGGAGGCAAGCGTTATCCGGAATTATTGGGCGTAAAGCGTCCGCAGGTGGTCTG<br>CCAAGTCTGCTGTCAAATCATGTTGCTTAACTACCTAAAGGCGGTGGAAACTGGCAGACTAGAGAGCATT<br>AGGGGTAGCAGGAATTCCCAGTGTAGCGGTGAAATGCGTAGATATTGGGAAGAACATCGGTGGCGAAAG<br>CGTGCTACTGGGCTGTATCTGACACTCAGGGACGAAAGCTAGGGGAGCGAAAGGGAT
BL1.29_781R	*Microcystis*	AATGGGCGAAAGCCTGACGGAGCAACGCCGCGTGAGGGAGGAAGGTCTTTGGATTGTAAACCTCTTTTCT<br>CAAGGAAGAAGTTCTGACGGTACTTGAGGAATCAGCCTCGGCTAACTCCGTGCCAGCAGCCGCGGTAATA<br>CGGGGGAGGCAAGCGTTATCCGGAATTATTGGGCGTAAAGCGTCCGCAGGTGGTCAGCCAAGTCTGCCGT

		CAAATCAGGTTGCTTAACGACCTAAAGGCGGTGGAAACTGGCAGACTAGAGAGCAGTAGGGGTAGCAGG<br>AATTCCCAGTGTAGCGGTGAAATGCGTAGAGATTGGGAAGAACATCGGTGGCGAAAGCGTGC
BL1.30_781R	*Microcystis*	CAATGGGCGAAAGCCTGACGGAGCAACGCCGCGTGAGGGAGGAAGGTCTTTGGATTGTAAACCTCTTTTC<br>TCAAGGAAGAAGTTCTGACGGTACTTGAGGAATCAGCCTCGGCTAACTCCGTGCCAGCAGCCGCGGTAAT<br>ACGGGGGAGGCAAGCGTTATCCGGAATTATTGGGCGTAAAGCGTCCGCAGGTGGTCAGCCAAGTCTGCC<br>GTCAAATCAGGTTGCTTAACGACCTAAAGGCGGTGGAAACTGGCAGACTAGAGAGCAGTAGGGGTAGCA<br>GGAAATTCCCAGTGTAGCGGTGAAATGCGTAGAGATTGGGAAGAAACATCGGTGGCGAAAAGCGTGC
BL4.35_359F	*Aphanizomenon*	GGCTCTTGGGTTGTAAACCTCTTTTCTCAGGGAAGAACAGAATGACGGTACCTGAGGAATAAGCATCGGC<br>TAACTCCGTGCCAGCAGCCGCGGTAATACGGAGGATGCAAGCGTTATCCGGAATGATTGGGCGTAAAGG<br>GTCCGCAGGTGGCATTGTAAGTCTGCTGTTAAAGAGTTTGGCTCAACCAAATAAGAGCAGTGGAAACTAC<br>AAAGCTAGAGTGTGGTCGGGGCAGAGGGAATTCCTGGTGTAGCGGTGAAATGCGTAGATATCAGGAAGA<br>ACACCAGTGGCGAAGGCGCTCTGCTAGGCCGAGACTGACACTGAGGGACGAAAGCTAGGGGAGCGAAT<br>GGGATTAGATACCCCAGTAGTCCTAGCCGTAAACGATGGATACTAGGCGTAGCTCGTATCGACCCGAGCT<br>GTGCCGGAGCTAACGCGTTAAGTATCCCGCCTGGGGAGTACGCAGGCAACTGTGAAACTCAAAGGAATT<br>GACGGGGGCCCGCACAAGCGGTGGAGTATGTGGTTTAATTCGATGCAACGCGAAGAACCTTACCAAGGC<br>TTGACATGTCACGAATCCTATTGAAAGATGGGAGTGCCTTCGGGAGCGTGAACACAGGTGGTGCATGGCT<br>GTCGTCAGCTCGTGTCGTGAGATGT
BL5.29_359F	*Dolichospermum*	TCCGATTATGTTCTACTTTCTCGGGAGCAGCTCTGTGGGATTGTAAACCTCTTTATCTCAGGGTAAGAAACA<br>ATGACGGTACGTGAGGAATAGGCATCGGCTAACTCCGTGCCAGCAGCCGCGGTAATACGGAGGATGCAA<br>GCGTTATCCGGAATGATTGGGCGTAAAGAGTCCGTAGGTGGCATTGAAAGTCTGCTGTTAAAGAGTCTAG<br>CTCAACTAGATAAGAGCAGTGGAAACTACAAAGCTAGAGTTTGGTCGGGGCAGAAGGAATTCCTGGTGT<br>AGCGGTGAAATGCGTAGATATCAGGAAGAACACCGGTGGCGAAGGCGTTCTGCTAGGCCGAGACTGACA<br>CTGAGGGACGAAAGCTAGGGGAGCGAATGGGATTAGATACCCCAGTAGTCCTAGCCGTAAACGATGGAT<br>ACTAGGCGTAGCTCGTATCGACCCGAGCTGTGCCGGAGCTAACGCGTTAAGTATCCCGCCTGGGGAGTAC<br>GCAGGCAACTGTGAAACTCAAAGGAATTGACGGGGGCCCGCACAAGCGGTGGAGTATGTGGTTTAATTC<br>GATGCAACGCGAAGAACCTTACCAAGGCTTGACATGTCACGAATCCTGTAGAAATATAGGAGTGCCTTCG<br>GGAGCGTGAACACAGGTGGTGCATGGCTGTCGTCAGCTCGTGTCGTGAGATG
BL5.35_359F	*Dolichospermum*	TCCGGAAGGGGCCGCACGGTCGCGTAGGACGCTCTGGCGTTCTCTCCTCTTTTCTCACGGAAGAAAAGAT<br>GACGGTACCTGAGGAATAGGCATCGGGTAACTCCGTGCCAGCAGCCGCGGTAATACGGAGGATGCAAGC<br>GTTATCCGGAATGATTGGGCGTAAAGGGTCCGCAGGTGGCATTGAAAGTCTGCTGTTAAAGAGTCTAGCT<br>CAACTAGATAAGAGCAGTGGAAACTACAAAGCTAGAGTTTGGTCGGGGCAGAAGGAATTCCTGGTGTAG<br>CGGTGAAATGCGTAGATATCAGGAAGAACACCAGTGGCGAAGGCGTTCTGCTAGGCCGAGACTGACACT<br>GAGGGACGAAAGCTAGGGGAGCGAATGGGATTAGATACCCCAGTAGTCCTAGCCGTAAACGATGGATAC<br>TAGGCGTAGCTCGTATCGACCCGAGCTGTGCCGGAGCTAACGCGTTAAGTATCCCGCCTGGGGAGTACGC<br>AGGCAACTGTGAAACTCAAAGGAATTGACGGGGGCCCGCACAAGCGGTGGAGTATGTGGTTTAATTCGA

		TGCAACGCGAAGAACCTTACCAAGGCTTGACATGTCACGAATCCTGTGGAAACATAGGAGTGCCTTCGGG<br>AGCGTGAACACAGGTGGTGCATGGCTGTCGTCAGCTCGTGTCGTGAGATGTTGGG
BL6.29_359F	*Dolichospermum*	AGGCTCTTGGGTTGTAAACCTCTTTTCTCAGGGAAGAAAAAAATGACGGTACCTGAGGAATAAGCATCGG<br>CTAACTCCGTGCCAGCAGCCGCGGTAATACGGAGGATGCAAGCGTTATCCGGAATGATTGGGCGTAAAG<br>GGTCCGCAGGTGGCATTGAAAGTCTGCTGTTAAAGAGTCTAGCTCAACTAGATAAGAGCAGTGGAAACTA<br>CAAAGCTAGAGTTTGGTCGGGGCAGAAGGAATTCCTGGTGTAGCGGTGAAATGCGTAGATATCAGGAAG<br>AACACCAGTGGCGAAGGCGTTCTGCTAGGCCGAGACTGACACTGAGGGACGAAAGCTAGGGGAGCGAAT<br>GGGATTAGATACCCCAGTAGTCCTAGCCGTAAACGATGGATACTAGGCGTAGCTCGTATCGACCCGAGCT<br>GTGCCGGAGCTAACGCGTTAAGTATCCCGCCTGGGGAGTACGCAGGCAACTGTGAAACTCAAAGGAATT<br>GACGGGGGCCCGCACAAGCGGTGGAGTATGTGGTTTAATTCGATGCAACGCGAA
BL7.30_359F	*Dolichospermum*	GGCTCTTGGGTTGTAACCTCTTTTCTCAGGGAAGAACAGAATGACGGTACCTGAGGAATAAGCATCGGCT<br>AACTCCGTGCCAGCAGCCGCGGTAATACGGAGGATGCAAGCGTTATCCGGAATGATTGGGCGTAAAGGG<br>TCCGCAGGTGGCATTGAAAGTCTGCTGTTAAAGAGTTTGGCTCAACCAAATAAGAGCAGTGGAAACTACA<br>AAGCTAGAGTTTGGTCGGGGCAGAGGGAATTCCTGGTGTAGCGGTGAAATGCGTAGATATCAGGAAGAA<br>CACCGGTGGCGAAGGCGCTCTGCTAGGCCGAGACTGACACTGAGGGACGAAAGCTAGGGGAGCGAATG<br>GGATTAGATACCCCAGTAGTCCTAGCCGTAAACGATGGATACTAGGCGTAGCTCGTATCGACCCGAGCTG<br>TGCCGGAGCTAACGCGTTAAGTATCCCGCCTGGGGAGTACGCAGGCAACTGTGAAACTCAAAGGAATTG<br>ACGGGGGCCCGCACAAGCGGTGGAGTATGTGGTTTAATTCGATGCAACGCGAAG
BL8.35_359F	*Microcystis*	GCCTCGGCTAACTCCGTGCCAGCAGCCGCGGTAATACGGGGGAGGCAAGCGTTATCCGGAATTATTGGGC<br>GTAAAGCGTCCGCAGGTGGTCAGCCAAGTCTGCCGTCAAATCAGGTTGCTTAACGACCTAAAGGCGGTGG<br>AAACTGGCAGACTAGAGAGCAGTAGGGGTAGCAGGAATTTCCCAGTGTAGCGGTGAAATGCGTAGAGAT<br>TGGGAAGAACATCGGTGGCGAAAGCGTGCTACTGGGCTGTATCTGACACTCAGGGACGAAAGCTAGGGG<br>AGCGAAAGGGAT
BV1.34_23S30R	*Synechococcus*	TATAGCATCGTTTCATTCCCAATTGTTTAATTTCAAACGTGGGTTGAAAAGACGCTGGGTTCACCAGATAC<br>TTGCATTTGAAGTGTAATTATTTGGAGATTCAGCAGAACCTTGACAACTGCATAGGTAAGTCTGGAAAGA<br>AAGCATCTCATAGATGTCCAGAAGCGAAAGCGAC
BV1.34_781R	*Microcystis*	TCCGTGCCAGCAGCCGCGGTAATACGGGGGAGGCAAGCGTTATCCGGAATCATTGGGCGTAAAGCGTCC<br>GCAGGTGGTCAGCCAAGTCAGCTGTCAAATCAGGTTGCTTAACGACATAAAGGCGGTGGAAACTGGCAG<br>ACTAGAGAGCAGTAGGGGTAGCGGGAATTCCCAGTGTAGCGGTGAAATGCGTAGAG
BV2.34_23S30R	*Microcystis*	AGCAAACCCGGCGTCAGTTCAGATTGCAGGCTGCAACTCGCCTGCATGAAGGAGGAATCGCTAGTAATCG<br>CCGGTCAGCATACGGCGGTGAATTCGTTCCCGGGCCTTGTACACACCGCCCGTCACACCATGGAAGCTGG<br>TCACGCCCGAAGTCATTACCTCAACCGCAAGGAGGGGGATGCCTAAGGCAGGGCTAGTGACTGGGGTGA<br>AGTCGTAACAAGGTAGCCGTACCGGAAGGTGTGGCTGGATCACCTCCTTAAAGGGAGACCTAATTCAGGT<br>ATAAGACGAAAAAAAAGTAGTCCCTACCAAGAATCAATCCCAAAAGGTCGGAACGAGGTATGAGGCTTT<br>CAAACTAGGTTCTGGGTTCATAAAAGACCTGAATCAGGAACAAGGGCTATTAGCTCAGGTGGTTAGAGCG

		CACCCCTGATAAGGGTGAGGTCCCTGGTTCGAGTCCAGGATGGCCCACCTGCACAGGTGGCAAAAACAA<br>GAGAAGCGAGGAATCAGCACCTTATCTTATATACATATATAAGAGAGAATGCTGGCTCTGAGT
BV2.35_359F	*Synechococcus*	TGTAACCTCTTTTCTCAAGGAAGAAGTTCTGACGGTACTTGAGGAATCAGCCACGGCTAATTCCGTGCCAG<br>CAGCCGCGGTAATACGGGGGTGGCAAGCGTTATCCGGAATCATTGGGCGTAAAGCGTCCGCAGGTGGCTT<br>TGTAAGTCTGCTGTTAAAGCGTGGAGCTTAACTCCCTTTCAGCGGTGGAAACTGCAAAACTTGAGTGTGGT<br>AGGGGCAGAGGGAATTCCCGGTGTAGCGGTGAAATGCGTAGATATCGGGAAGAACACCAGTGGCGAAG<br>GCGCTCTGCTGGGCCATATCTGACACTCATGGACGAAAGCCAGGGGAGCGAAAGGGATTAGATACCCCT<br>GTAGTCCTGGCCGTAAACGATGAACACTAGGCGTCGGGGGAATCGACCCCCTCGGTGTCGTAGCCAACGC<br>GTTAAGTGTTCCGCCTGGGGAGTACGCACGCAAGTGTGAAACTCAAAGGAATTGACGGGGGCCCGCACA<br>AGCGGTGGAGTATGTGGTTTAATTCGATGCAACGCGAAGAACCTTACCAGACTTGACA
BV3.35_359F	*Phormidium*	TCCGCTTAACAACACAGCCGCTCAGGCTATGTACGTTCTGTGGATGTAACCTCTTTTCTCAGGGAAGACAT<br>CTGACGGTACCTGAGGAATAAACGTCGAAGTAACTCCGTGCCAGCAGCCGCGGTAATACGGAGGATGCA<br>AGCGTTATCCGGAATGATTGGGCGTAAAGCGTCCGCAGGTGGTTTTTCAAGTCTGCTGTTAAAGACCGGG<br>GCTTAACTCCAGGCAAGCAGTGGAAACTGAAAGACTAGAGTATGGTAGGGGCAGAGGGAATTCCTGGTG<br>TAGCGGTGAAATGCGTAGAGCTCAGGAAGAACATCGGTGGCGAAGGCGCTCTGCTAGGCCGAAACTGAC<br>ACTCAGGGACGAAAGCTAGGGGAGCGAATGGGATTAGATACCCCAGTAGTCCTAGCTGTAAACGATGGA<br>TACTAGGTGTTGTCTGTATCGACCCGAACTGTGCCGTAGCTAACGCGTTAAGTATCCCGCCTGGGGAGTAC<br>GCACGCAAGTGTGAAACTCAAAGGAATTGACGGGGGCCCGCACAAGCGGTGGAGTATGTGGTTTAATTC<br>GATGCAACGCGA
B04.27_359F	*Aphanizomenon*	CTTTTCTCAGGGAAGAACACAATGACGGTACCTGAGGAATAAGCATCGGCTAACTCCGTGCCAGCAGCCG<br>CGGTAATACGGGGGATGCAAGCGTTATCCGGAATGATTGGGCGTAAAGGGTCCGCAGGTGGCATTGCAA<br>GTCTGCTGTTAAAGAGTTTGGCTCAACCTCATAAAAGCAGTGGAAACTGCAAAGCTAGAGTGTGGTCGGG<br>GCAGAGGGAATTCCTGGTGTAGCGGTGAAATGCGTAGAGATCAGGAAGAACACCGGTGGCGAAGGCGCT<br>CTGCTAGGCCATAACTGACACTGAGGGACGAAAGCTAGGGGAGCGAATGGGATTAGATACCCCAGTAGT<br>CCTAGCCGTAAACGATGGATACTAGGCGTGGCTCGTATCGACCCGAGCTGTGCCGTAGCTAACGCGTTAA<br>GTATCCCGCCTGGGGAGTACGCACGCAACTGTGAAACTCAAAGGAATTGACGGGGGCCCGCACAAGCGG<br>TGGAGTATGTGGTTTAATTCGATGCAACGCGAAGAACCTTACCAAGGCTTGACATCTC
B04.29_359F	*Aphanizomenon*	TACGGAGGATGCAAGCGTTATCCGGAATGATTGGGCGTAAAGGGTCCGCAGGTGGCATTGTAAGTCTGCT<br>GTTAAAGAGTTTGGCTCAACCAAATAAAAGCAGTGGAAACTACAAAGCTAGAGTGTGGTCGGGGCAGAG<br>GGAATTCCTGGTGTAGCGGTGAAATGCGTAGATATCAGGAAGAACACCGGTGGCGAAGGCGCTCTGCTA<br>GGCCAAGACTGACACTGAGGGACGAAAGCTAGGGGAGCGAATGGGATTAGATACCCCAGTAGTCCTAGC<br>CGTAAACGATGGATACTAGGCGTAGCTCGTATCGACCCGAGCTGTGCCGTAGCTAACGCGTTAAGTATCC<br>CGCCTGGGGAGTACGCAGGCAACTGTGAAACTCAAAGGAATTGACGGGGGCCCGCACAAGCGGTGGAGT<br>ATGTGGTTTAATTCGATGCAACGCGA
B04.29_781R	*Planktothrix*	CGGAGCAAGACCGCGTGGGGGAGGAAGGTTCTTGGATTGTCAACCCCTTTTTTCAGGGAAGAACACAATG<br>ACGGTACCTGAGGAATAAGCATCGGCTAACTCCGTGCCAGCAGCCGCGGTAATACGGGGGATGCAAGCG

		TTATCCGGAATGATTGGGCGTAAAGAGTCCGTAGGTGGTCATCCAAGTCTGCTGTTAAAGAGCGAGGCTT<br>AACCTCGTAAAGGCAGTGGAAACTGGAAGACTAGAGTGTAGTAGGGGCAGAGGGAATTCCCGGTGTAGC<br>GGTGAAATGCGTAGATATCAGGAAGAACACCGGTGGCGA
B04.31_359F	*Aphanizomenon/Dolichospermum*	GTTGTAAACCTCTTTTCTCAGGAAGAACATCTTGCGGGTCTTGGAGGATTAACCATCGGTTATTTCCGGGC<br>CACCACCCGGGGAATTCCGGAGGGTGCAGCGTTTTTCGGGATTGTTGGGGGGTAAGGGGCCCGCAGGGG<br>GCTTTGTAGTTCGGCTGTTAAACATGTTGCTTCACTCCATTTCAGCGATGGAAACTACAAGGCTAGAGTGT<br>GGTCGGGGCAGAGGGAATTCCTGGTGTAGCGGTGAAATGCGTAGATATCAGGAAGAACACCGGTGGCGA<br>AGGCGCTCTGCTGGGCCATAACTGACACTGATGGACGAAAGCTAGGGGAGCGAATGGGATTAGATACCC<br>CTGTAGTCCTGGCCGTAAACGATGAATACTAGGCGTAGCTCGAATCGACCCCCTCTGTGTCGTAGCTAACG<br>CGTTAAGTGTTCCGCCTGGGGAGTACGCACGCAACTGTGAAACTCAAAGGAATTGACGGGGGCCCGCAC<br>AAGCGGTGGAGTATGTGGTTTAATTCGATGCAACGCGA
B04.34_359F	*Aphanizomenon*	CTTGGGTTGTAACCTCTTTTCTCAGGGAAGAACAAATTGACGGTACCTGAGGAATAACCATCGGCTAATTC<br>CGGGCCACCAGCCGCGGTAATACGGAGGATGCAAGCGTTATCCGGAATGATTGGGCGTAAAGGGTCCGC<br>AGGGGGCATTGTAATTCTGCTGTTAAAGAGTTTGGCTCAACCAAATAAAAGCAATGGAAACTACAAAGCT<br>AGAGTGTGGTCGGGGCAGAGGGAATTCCTGGTGTAGCGGTGAAATGCGTAGATATCAGGAAGAACACCG<br>GTGGCGAAGGCGCTCTGCTAGGCCAAGACTGACACTGAGGGACGAAAGCTAGGGGAGCGAATGGGATTA<br>GATACCCCAGTAGTCCTAGCCGTAAACGATGGATACTAGGCGTAGCTCGAATCGACCCGAGCTGTGCCGT<br>AGCTAACGCGTTAAGTATCCCGCCTGGGGAGTACGCACGCAACTGTGAAACTCAAAGGAATTGACGGGG<br>GCCCGCACAAGCGGTGGAGTATGTGGTTTAATTCGATGCAACGCGAAGAACCTTACCAAGGCTTGACATG<br>TCACGAATTCCGTTGAA
E04.29_359F	*Aphanizomenon*	CTCTTGGGTTGTAACCTCTTTTCTCAGGGAAGAACAGAATGACGGTACCTGAGGAATAAGCATCGGCTAA<br>CTCCGTGCCAGCAGCCGCGGTAATACGGAGGATGCAAGCGTTATCCGGAATGATTGGGCGTAAAGGGTCC<br>GCAGGTGGCATTGTAAGTCTGCTGTTAAAGAGTTTGGCTCAACCAAATAAGAGCAGTGGAAACTACAAAG<br>CTAGAGTGTGGTCGGGGCAGAGGGAATTCCTGGTGTAGCGGTGAAATGCGTAGATATCAGGAAGAACAC<br>CAGTGGCGAAGGCGCTCTGCTAGGCCGAGACTGACACTGAGGGACGAAAGCTAGGGGAGCGAATGGGA<br>TTAGATACCCCAGTAGTCCTAGCCGTAAACGATGGATACTAGGCGTAGCTCGTATCGACCCGAGCTGTGC<br>CGGAGCTAACGCGTTAAGTATCCCGCCTGGGGAGTACGCAGGCAACTGTGAAACTCAAAGGAATTGACG<br>GGGGCCCGCACAAGCGGTGGAGTATGTGGTTTAATTCGATGCAACGCGAAGAACCTTACCAAGGCTTGAC<br>ATGTCACGAATCCTATTGAAAGATGGGAGTGCCTTCGGGAGCGTGAACACAGGTGGTGCATGGCTGTCGT<br>CAGCTCGTGTCGTGAGATGTTGG
E04.32_781R	rhodoplast	TCTTCCTATCTCTACGCATTTCACCGCTACACTGGGAATTCCCTCTACCTCTACTGCACTCTAGTCTGCTAGT<br>TTCCACCGCCTTTATGACGTTAAGCCCCGAGATTTAACAGCAGACTTGGTAGGCCACCTACAGACGCTTTA<br>CGCCCAGTGATTCCGGATAACGCTTGCATCCTCCGTATTACCGCGGCTGCTGGCACGGAGTTAGCCGATGC<br>TTATTCCTCAAGTACCGTCAGTACTTCTTCC
E04.34_359F	*Microcystis*	CTTGAGGTACTTGAGGAACAGCCTCGGCTCACTCGTGCCAGCAGCCGCGGTAATACGGGGGAGGCAAGC<br>GTTTTCCGGAATTATTGGGCGTAAAGCGTCCGCAGGTGGTCTTCCAAGTCTGCCGTCAAATCACGTTGCTT

		AACCACCTAAAGGCGGTGGAAACTGGCAGACTAGAGAGCAGTAGGGGTAGCAGGAATTCCCAGTGTAGC<br>GGTGAAATGCGTAGAGATTGGGAAGAACATCGGTGGCGAAAGCGTGCTACTGGGCTGTATCTGACACTC<br>AGGGACGAAAGCTAGGGGAGCGAAAGGGATA
E04.36_359F	*Microcystis*	GTACTGAGGAAAAGCCTGGCTAACTCTGTGCCGCAGCCGCGGTAAACGGGGGAGGCAAGCGTTTTTCCGG<br>ATTATTGGGCGTAAAGCGTCCGCAGGTGGTCTGCCAAGTCTGCCGTCAAATATCGTTGCTTAACCACCTAA<br>AGGCGGTGGAAATGGCAGACTAGAGTGCAGTAGGGGTAGCAGGAATTCCCAGTGTAGCGGTGAAATGCG<br>TAATATTGGGAAGAACATCGGTGGCGAAAGCGTGCTACTGGGCTGTATCTGACACTCAGGGACGAAAGCT<br>AGGGGAGCG
H02.27_359F	*Synechococcus*	GCCTCTGGGCTGTAAACCTCTTTTCTCAAGGAAGAAGATCTGACGGTACTTGAGGAATAAGCCACGGCTA<br>ATTCCGTGCCAGCAGCCGCGGTAATACGGGAGTGGCAAGCGTTATCCGGAATTATTGGGCGTAAAGCGTC<br>CGCAGGCGGCCTTGTAAGTCTGCCGTTAAAGCGTGGAGCTTAACTCCATTTCGGCGATGGAAACTACAAG<br>GCTTGAGTGTGGTAGGGGCAGAGGGAATTCCCGGTGTAGCGGTGAAATGCGTAGATATCGGGAAGAACA<br>CCAGTGGCGAAGGCGCTCTGCTGGGCCATAACTGACGCTCATGGACGAAAGCCAGGGGAGCGAAAGGG<br>ATTAGATACCCCTGTAGTCCTGGCCGTAAACGATGAACACTAGGTGTCGGGGGAATCGACCCCCTCGGTG<br>TCGTAGCCAACGCGTTAAGTGTTCCGCCTGGGGAGTACGCACGCAAGTGTGAAACTCAAAGGAATTGACG<br>GG
H02.31_359F	*Planktothrix*	TGCGTTGGAAGGGTGGGCTGGAGGGAGAGCCTCTTGGGACTGTCAACCTCTTTTCTCAGGGAAGAACACC<br>ATGACGGTCCCTGAGGATAAAGCATCGGCTAACTCCGTGCCAGCACCCGCGGTAATACGGGGGATGCAA<br>GCGTTATCCGGAATGATTGGGCGTAAAGAGTCCGTAGGTGGTCATCCAAGTCTGCTGTTAAAGATGAAGC<br>TTAACCTCTTTCGGCAGTGGAAACTGGAAGATAGAGTGTAGTAGGGGCAGAGGGAATTCCTGGTGTAGCG<br>GTGAAATGCGTAGAGTCAGGAAGAACACCGGTGGCGAAGGCGCTCTGCTGGGCTATAACTGACACTGAG<br>GGCGAAAGCTAGGGGAGCGAATGGGATTAGATACCCC
H02.32_359F	*Aphanizomenon*	AACCTCTTTTCTCAGGGAAGAAAAACACGACGGTACCTTGAGGAATAAGCATCGGCTAACTCCGTGCCAG<br>CAGCCGCGGTAATACGGAGGATGCAAGCGTTATCCGGAATGATTGGGCGTAAAGGGTCCGCAGGTGGTA<br>GTGTAAGTCTGCTGTTAAAGAATCACGCTCAACGTGATCAAAGCAGTGGAAACTACACAACTAGAGTACG<br>GTAGGGGCAGAAGGAATTCCTGGTGTAGCGGTGAAATGCGTAGATATCAGGAAGAACACCGGTGGCGAA<br>AGCGTTCTGCTAGACCTGTACTGACACTGAGGGACGAAAGCTAGGGGAGCGAATGGGATTAGATACCCC<br>AGTAGTCCTAGCCGTAAACGATGGATACTAGGTGTGGCTTGTATCGACCCGAGCCGTACCGTAGCTAACG<br>CGTTAAGTATCCCGCCTGGGGAGTACGCACGCAAGTGTGAAACTCAAAGGATTGACGGG
H02.35_359F	*Planktothrix*	CCGTGCCAGCAGCCGCGGTAATACGGGGGATGCAAGCGTTATCCGGAATGATTGGGCGTAAAGAGTCCG<br>TAGGTAGTCATCCAAGTCTGCTGTTAAAGAGCGAGGCTTAACCTCGTAAAGGCAGTGGAAACTGGAAGAC<br>TAGAGTGTAGTAGGGGCAGAGGGAATTCCTGGTGTAGCGGTGAAATGCGTAGAGATCAGGAAGAACACC<br>GGTGGCGAAGGCGCTCTGCTGGGCTATAACTGACACTGAGGGACGAAAGCTAGGGGAGCGAATGGGATT<br>AGATACCCCAGTAGTCCTAGCGGTAAACGATGGAAACTAGGTGTGGCCTGTATCGACCCGGGCCGTGCCG<br>AAGCAAACGCGTTAAGTTTCCCGCCTGGGGAGTACGCACGCAAGTGTGAAACTCAAAGGAATTGACGGG

		GGCCCGCACAAGCGGTGGAGTATGTGGTTTAATTCGATGCAACGCGAAGAACCTTACCAGGACTTGACATCTCTGGAATCTC
H02.36_359F	*Synechococcus*	CTCTGGGCTGTAACCTCTTTTCTCAAGGAAGAAGATCTGACGGTACTTGATGAATAAGCCACGGCTAATTCCGTGCCAGCAGCCGCGGTAATACGGGAGTGGCAAGCGTTATCCGGAATTATTGGGCGTAAAGCGTCCGCAGGCGGCCTTGTAAGTCTGTCGTTAAAGCGTGGAGCTTAACTCCATTTCAGCGATGGAAACTGCAAGGCTTGAGTGTGGTAGGGGCAGAGGGAATTCCCGGTGTAGCGGTGAAATGCGTAGATATCGGGAAGAACACCAGTGGCGAAGGCGCTCTGCTGGGCCATAACTGACGCTCATGGACGAAAGCCAGGGGAGCGAAAGGGATTAGATACCCCTGTAGTCCTGGCCGTAAACGATGAACACTAGGTGTCGGGGGAATCGACCCCCTCGGTGTCGTAGCCAACGCGTTAAGTGTTCCGCCTGGGGAGTACGCACGCAAGTGTGAAACTCAAAGGAATTGACGGGGGCCCGCACAAGCGGTGGAGTATGTGGTTTAATTCGATGCAACGCGAAGAACCTTAC
I01.28_781R	*Cyanobium*	ACCGCTACACCGGGAATTTCCTCTGCCCTACCACACTCTAGTTCTACAGTTTCCATCGCTGAAATGGAGTTAAGCTCCACGTTTTAACGACAGACTTGTAAAACCGCCTGCGGACGCTTTACGCCCAATAATTCCGGATAACGCTTGCCACTCCCGTATTACCGCGGCTGCTGGCACGGAATTAGCCGAGGCTTATTCATCAAGTACCGTCAGATCTTCTTCCTTGATAAAAGAGGTTTACAGCCCACAGGCCTTCATCCCTCACGCGGCGTTGCTCCGTCAGGCTTTC
I01.35_359F	*Synechococcus*	AGGCCTCTGGGCTGTAAACCTCTTTTCTCAAGGAAGAAGATCTGACGGTACTTGAGGAATAAGCCACGGCTAATTCCGTGCCAGCAGCCGCGGTAATACGGGAGTGGCAAGCGTTATCCGGAATTATTGGGCGTAAAGCGTCCGCAGGCGGCCTTGTAAGTCTGTCGTTAAAGCGTGGAGCTTAACTCCATTTCAGCGATGGAAACTACAAGGCTTGAGTGTGGTAGGGGCAGAGGGAATTCCCGGTGTAGCGGTGAAATGCGTAGATATCGGGAAGAACACCAGTGGCGAAGGCGCTCTGCTGGGCCATAACTGACGCTCATGGACGAAAGCCAGGGGAGCGAAAGGGATTAGATACCCCTGTAGTCCTGGCCGTAAACGATGAACACTAGGCGTCGGGGGAATCGACCCCCTCGGTGTCGTAGCCAACGCGTTAAGTGTTCCGCCTGGGGAGTACGCACGCAAGTGTGAAACTCAAAGGAATTGACGGGGGCCCGCACAAGCGGTGGAGTATGTGGTTTAATTCGATGCAACGCGAAGA
I01.37_781R	*Cyanobium*	ATGGGCGAAAGCCTGACGGAGCAACGCCGCGTGAGGGATGAAGGCCTGTGGGCTGTAAACCTCTTTTCTCAAGGAAGAAGATCTGACGGTACTTGAGGAATAAGCCACGGCTAATTCCGTGCCAGCAGCCGCGGTAATACGGGAGAGGCAAGCGTTATCCGGAATCATTGGGCGTAAAGCGTCCGCAGGCGGCCTTGCAAGTCTGTCGTTAAAGCGTGGAGCTTAACTCCATAAAAGCGGTGGAAACTACAAGGCTAGAGTGTGGTAGGGGCAGAGGGAATTCCCGGTGTAGCGGT
I04.27_359F	*Aphanizomenon*	GCTCTTGGGTTGTAAACCTCTTTTCTCAGGGAAGAACAAAATGACGGTACCTGAGGAATAAGCATCGGCTAATTCCGTGCCAGCAGCCGCGGTAATACGGAGGATGCAAGCGTTATCCGGAATGATTGGGCGTAAAGGGTCCGCAGGTGGCATTGTAAGTCTGCTGTTAAAGAGTTTGGCTCAACCAAATAAGAGCAGTGGAAACTACAAAGCTAGAGTGTGGTCGGGGCAGAGGGAATTCCTGGTGTAGCGGTGAAATGCGTAGATATCAGGAAGAACACCGGTGGCGAAGGCGCTCTGCTAGGCCAAAACTGACACTGAGGGACGAAAGCTAGGGGAGCGAATGGGATTAGATACCCCAGTAGTCCTAGCCGTAAACGATGGATACTAGGCGTAGCTCGTATCGACCCGAGCTGTGCCGTAGCTAACGCGTTAAGTATCCCGCCTGGGGAGTACGCAGGCAACTGTGAAACTCAAAGGAATTGACGGGGGCCCGCACAAGCGGTGGAGTATGTGGTTTAATTCGATGCAACGCGAAGAACCTTACCAAGG

I04.28_359F	*Aphanizomenon*	GCTCTTGGGTTGTAACCTCTTTTCTCAGGGAAGAACAACATGACGGTACCTGAGGAATAAGCATCGGCTA<br>ACTCCGTGCCAGCAGCCGCGGTAATACGGAGGGTGCAAGCGTTATCCGGAATGATTGGGCGTAAAGGGT<br>CCGCAGGTGGCATTGTAAGTCTGCTGTTAAAGAGTTTGGCTCAACCAGATAAGAGCAGTGGAAACTACAA<br>AGCTAGAGTGTGGTCGGGGCAGAGGGAATTCCTGGTGTAGCGGTGAAATGCGTAGATATCAGGAAGAAC<br>ACCGGTGGCGAAGGCGCTCTGCTAGGCCAAGACTGACACTGAGGGACGAAAGCTAGGGGAGCGAATGG<br>GATTAGATACCCCAGTAGTCCTAGCCGTAAACGATGGATACTAGGCGTAGCTCGTATCGACCCGAGCTGT<br>GCCGTAGCTAACGCGTTAAGTATCCCGCCTGGGGAGTACGCAGGCAACTGTGAAACTCAAAGGAATTGA<br>CGGGGGCCCGCACAAGCGGTGGAGTATGTGGTTTAATTCGATGCAACGCGAAGAACCTTACCAAGGCTTG<br>ACATGTCACGAATTCTGTTGAAATATGGGAGTGCCTTCGGGAGCGTGAACACA
I04.29_359F	*Dolichospermum*	CTTGGGTTGTAAACCTCTTTTCTCAGGGAAGAACAGAATGACGGTACCTGAGGAATAAGCATCGGCTAAC<br>TCCGTGCCAGCAGCCGCGGTAATACGGAGGATGCAAGCGTTATCCGGAATGATTGGGCGTAAAGGGTCC<br>GCAGGTGGCATAGTAAGTCTGCTGTTAAAGAGTCACGCTCAACGTGATAAGAGCAGTGGAAACTACAAA<br>GCTAGAGTATGGTCGGGGCAGAGGGAATTCCTGGTGTAGCGGTGAAATGCGTAGATATCAGGAAGAACA<br>CCGGTGGCGAAGGCGCTCTGCTAGGCCAAAACTGACACTGAGGGACGAAAGCTAGGGGAGCGAATGGG<br>ATTAGATACCCCAGTAGTCCTAGCCGTAAACGATGGATACTAGGCGTAGCTCGTATCGACCCGAGCTGTG<br>CCGTAGCTAACGCGTTAAGTATCCCGCCTGGGGAGTACGCAGGCAACTGTGAAACTCAAAGGAATTGAC<br>GGGGGCCCGCACAAGCGGTGGAGTATGTGGTTTAATTCGATGCAACGCGAAGAACCTTACCAAGGCTTGA<br>CATGTCACGAATCCTGTAGAAATATAGGAGTGCC
I04.30_359F	*Dolichospermum*	GGCTCTTGGGTTGTAACCTCTTTTCTCAGGGAAGAACAGAATGACGGTACCTGAGGAATAAGCATCGGCT<br>AACTCCGTGCCAGCAGCCGCGGTAATACGGAGGATGCAAGCGTTATCCGGAATGATTGGGCGTAAAGGG<br>TCCGCAGGTGGCATAGTAAGTCTGCTGTTAAAGAGTCTGGCTCAACGAGATAAGAGCAGTGGAAACTACA<br>AAGCTAGAGTTTGGTCGGGGCAGAGGGAATTCCTGGTGTAGCGGTGAAATGCGTAGATATCAGGAAGAA<br>CACCGGTGGCGAAGGCGCTCTGCTAGGCCAAAACTGACACTGAGGGACGAAAGCTAGGGGAGCGAATG<br>GGATTAGATACCCCAGTAGTCCTAGCCGTAAACGATGGATACTAGGCGTAGCTCGTATCGACCCGAGCTG<br>TGCCGTAGCTAACGCGTTAAGTATCCCGCCTGGGGAGTACGCAGGCAACTGTGAAACTCAAAGGAATTGA<br>CGGGGGCCCGCACAAGCGGTGGAGTATGTGGTTTAATTCGATGCAACGCGAAGAACCTTACCAAGGCTTG<br>ACATGTCACGAATCCTGTAGAAATATAGGAGTGCCT
I04.31_359F	*Dolichospermum*	CTTGGGTTGTAAACCTCTTTTCTCAGGGAAGAACAGAATGACGGTACCTGAGGAATAAGCATCGGCTAAC<br>TCCGTGCCAGCAGCCGCGGTAATACGGAGGATGCAAGCGTTATCCGGAATGATTGGGCGTAAAGGGTCC<br>GCAGGTGGCATAGAAAGTCTGCTGTTAAAGAGTCTGGCTCAACCTGATAAGAGCAGTGGAAACTACAAA<br>GCTAGAGTATGGTCGGGGCAGAGGGAATTCCTGGTGTAGCGGTGAAATGCGTAGATATCAGGAAGAACA<br>CCGGTGGCGAAGGCGCTCTGCTAGGCCAAAACTGACACTGAGGGACGAAAGCTAGGGGAGCGAATGGG<br>ATTAGATACCCCAGTAGTCCTAGCCGTAAACGATGGATACTAGGCGTAGCTCGTATCGACCCGAGCTGTG<br>CCGTAGCTAACGCGTTAAGTATCCCGCCTGGGGAGTACGCAGGCAACTGTGAAACTCAAAGGAATTGAC<br>GGGGGCCCGCACAAGCGGTGGAGTATGTGGTTTAATTCGATGCAACGCGAAGAACCTTACCAAGGCTTGA

		CATGTCACGAATCCTGTAGAAATATAGGAGTGCCTTAGGGAGCGTGAACACAGGTGGTGCATGGCTGTCGTCAGCTCGTGTCGTGAGATGTTGGGTTAAGTCC
I04.32_359F	*Microcystis*	ATCAGCCTCGGCTAACTCCGTGCCAGCAGCCGCGGTAATACGGGGGAGGCAAGCGTTATCCGGAATTATTGGGCGTAAAGCGTCCGCAGGTGGTCATCCAAGTCTGCCGTCAAATCAGGTTGCTTAACGACCTAAAGGCGGTGGAAACTGGCAGACTAGAGAGCAGTAGGGGTAGCAGGAATTCCCAGTGTAGCGGTGAAATGCGTAGAGATTGGGAAGAACATCGGTGGCGAAAGCGTGCTACTGGGCTGTATCTGACACTCAGGGACGAAAGCTAGGGGAGCGAAAGGG
I04.32_359F	*Dolichospermum*	CTGAGGAATAAGCATCGGCTAACTCCGGGCCACCAGCCGCGGTAATACGGAGGATGCAAGCGTTATCCGGAATGATTGGGCGTAAAGGGTCCGCAGGTGGCATTGAAAGTCTGCTGTTAAAGAGTCTGGCTCAACCTGATAAGAGCAGTGGAAACTACAAAGCTAGAGTTTGGTCGGGGCAGAGGGAATTCCTGGTGTAGCGGTGAAATGCGTAGATATCAGGAAGAACACCGGTGGCGAAGGCGCTCTGCTAGGCCAAACTGACACTGAGGGACGAAAGCTAGGGGAGCGAATGGGATTAGATACCCCAGTAGTCCTAGCCGTAAACGATGGATACTAGGCGTAGCTCGTATCGACCCGAGCTGTGCCGGAGCTAACGCGTTAAGTATCCCGCCTGGG
I04.34_359F	*Microcystis*	GGAATTCAGCCTCGGCTAACTTCCGTGCCAGCAGCCGCGGTAATACGGGGGAGGCAAGCGTTTATCCGGAATTATTGGGCGTAAAGCGTCCGCAGGTGGTCAGCCAAGTCTGCCGTCAAATCAGGTTGCTTAACCACCTAAAGGCGGTGGAAACTGGCAGACTAGAGAGCAGTAGGGGTAGCAGGAATTCCCAGTGTAGCGGTGAAATGCGTAGAGATTGGGAAGAACATCGGTGGCGAAAGCGTGCTACTGGGCTGTATCTGACACTCAGGGACGAAAGCTAGGGGAGCGAAAGGG
I04.36_359F	*Planktothrix*	AGTCTGCTGTTAAAGAGGGAGGCTTAACCTCTTTCGGCAGTGGAAACTGGAAGATAGAGTGTGGTAGGGGCAGAGGGAATTCCTGGTGTAGCGGTGAAATGCGTAGAGTCAGGAAGAACACCGGTGGCGAAGGCGCTCTGCTGGGCTATAACTGACACTGAGGGACGAAAGCTAGGGGAGCGAATGGGTTAGATACCCCAGTAGTCCTAGCGGAAACGATGGAACT
I04.37_359F	*Cyanodictyon*	GGAGCTTAACTCCATTTCAGCGGTGGAAACTACAAAGCTTGAGTGTGGTAGGGGCAGAGGGAATTCCCGGTGTAGCGGTGAAATGCGTAGATATCGGGAAGAACACCAGTGGCGAAGGCGCTCTGCTGGGCCATAACTGACACTCATGGACGAAAGCCAGGGGAGCGAATGGGATTAGATACCCCTGTAGTCCTGGCCGTAAACGATGAACACTAGG
